# The Pan-American Medical Congress

**Published:** 1893-10

**Authors:** 


### EDITORIAL.

### The Pan-American Medical Congress.

This great convocation of medical men from all American countries, for which preparation has been actively going on for two years, was held in Washington, D. C., September 5th to 9th inclusive.

The meeting was in some respects the most notable that has ever been held in this country; in it were brought together the leading men of nearly all the American countries, of all, indeed, who were in a condition to send delegates. There were registered about eleven hundred members.

The object of this organization was to bring together the members of the medical profession of the Western Continent who had, in the main, hitherto been almost total strangers. Physicians of American States outside of the United States had much less acquaintance with each other than both had with the physicians of European countries. In the past very slight intercourse indeed has existed between the physicians of the different American countries, and largely because those of these different States or countries have had more intercourse with the profession of Europe, especially with England and France, than they had with those of the United States. The large proportion of American students from the American States or countries, outside of the United States, went to European colleges for their professional education, and on this account very little contact was had between these American countries and the United States. It is to be hoped, however, that in the future this will be changed, in a good degree, at least.

The papers read and the work done by this congress were of very great interest; they looked towards a closer affiliation and intercourse by the profession in these different countries. Here, as in most other gatherings of medical men for the past three or four years, much consideration was given to sanitary science. A great and much desired change is manifestly taking place in the medical profession on this subject. It is being more and more brought into the curriculum of the medical colleges, and it will be, no doubt, in the near future, as it ought to be, one, if not the leading branch for instruction in the medical colleges. In this congress was advocated very strongly the propriety and importance of making the public health a department in general governmental affairs, and, indeed, in every place where legislation can be brought to bear upon the subject. Methods of quarantine involving the exclusion of disease were fully discussed.

The special work of the congress was done in sections, of which there were twenty-one in number. Prominent among these was the section of Hygiene, Climatology and Quarantine. The organization of Pedagogy was comparatively a new movement, but great interest was taken in it. Bringing this subject into so prominent a position, as was done here, must necessarily in the near future result in securing more uniformity, and the adoption of the best methods of teaching in the different medical colleges of the world. The members of faculties of medical colleges who were present took very great interest in the work of this section, and it is a subject that, without doubt, will in the future have a far more prominent position before the medical world.

There was also a section on Oral and Dental Surgery, which in the organization occupied the same position as the other sections. Quite a number of most excellent papers were read and discussed; several papers were presented, for the reading and consideration of which there was not time, so that they could only be read by title. Upon the whole the occasion was an exceedingly interesting one, and especially so, when the results and future possibilities are considered.

For the first suggestion and the setting in motion the influences that resulted in this great meeting, and for planning, arranging and carrying out the arrangements for its completion, the medical profession is largely indebted to Dr. Charles A. L. Reed, Secretary General of the Congress, of Cincinnati, Ohio, who has executed the great work devolving upon this position in a most masterly manner.

The congress adjourned to meet in the City of Mexico in 1896 or 1897.

